# Artesunate Regulates Neurite Outgrowth Inhibitor Protein B Receptor to Overcome Resistance to Sorafenib in Hepatocellular Carcinoma Cells

**DOI:** 10.3389/fphar.2021.615889

**Published:** 2021-02-25

**Authors:** Wubin He, Xiaoxu Huang, Bradford K. Berges, Yue Wang, Ni An, Rongjian Su, Yanyan Lu

**Affiliations:** ^1^ Key laboratory of surgery of Liaoning Province of The First Affiliated Hospital of Jinzhou Medical University, Liaoning, China; ^2^ Key Laboratory of Molecular Cell Biology and New Drug Development of Jinzhou Medical University, Liaoning, Jinzhou, China; ^3^ Department of Microbiology and Molecular Biology of Brigham Young University, Provo, UT, United States; ^4^ Department of Pathlogy of The First Affiliated Hospital of Jinzhou Medical University, Liaoning, China; ^5^ Department of Orthopedic Spine Surgery of The First Affiliated Hospital of Jinzhou Medical University, Liaoning, China

**Keywords:** artesunate, NGBR, sorafenib, resistance, hepatocellular carcinoma

## Abstract

The multireceptor tyrosine kinase inhibitor sorafenib is a Food and Drug Administration-approved first-line drug for the treatment of advanced liver cancer that can reportedly extend overall survival in patients with advanced hepatocellular carcinoma (HCC). Primary and acquired resistance to sorafenib are gradually increasing however, leading to failure of HCC treatment with sorafenib. It is therefore crucial to study the potential mechanism of sorafenib resistance. The results of the current study indicate that neurite outgrowth inhibitor protein B receptor (NgBR) is overexpressed in cultured sorafenib-resistant cells, and that its expression is negatively correlated with the sensitivity of liver cancer cells to sorafenib. Artesunate can inhibit the expression of NgBR, and it may block sorafenib resistance. Herein we report that sorafenib treatment in combination with artesunate overcomes HCC resistance to sorafenib alone in a cell culture model.

## Introduction

Hepatocellular carcinoma (HCC) is a primary solid tumor that develops chemoresistance. This limits the number of effective interventions for advanced HCC, leading to over 700,000 deaths annually around the world (Fukui et al., 2018). In early stages HCC can be treated using tumor resection, ablation, or liver transplantation (Díaz-González et al., 2020). The successful development of molecularly targeted drugs has reportedly extended the overall survival of patients with chronic liver cancer (Al-Salama et al., 2019).

The multityrosine kinase inhibitor sorafenib was the first small-molecule-targeted drug that demonstrably extended the overall survival of advanced HCC patients (Gramantieri et al., 2020). In the last decade it has been widely used as the first-line therapeutic drug for the treatment of chronic HCC (Wang et al., 2020). Most patients with advanced HCC develop resistance to sorafenib early during treatment, and thus do not benefit from it in the long term (Zhang et al., 2020). A previous report has demonstrated the role of mitogen-activated protein kinase 14 (MAPK14) activation in the development of sorafenib resistance in HCC (Rudalska et al., 2014). MAPK14 activation in HCC cells was associated with no response to sorafenib in a murine model of tumor development and treatment with sorafenib (Hashiba et al., 2020). In other studies persistent activation of mitogen-activated protein kinase (MAPK kinase)/extracellular signal-regulated kinase (MEK/ERK) signaling pathways was evident in HCC due to the development of sorafenib resistance (Sung et al., 2018; Ghousein et al., 2020).

Artesunate is one of the derivatives of artemisinin, and it can inhibit tumor cell proliferation, tumor angiogenesis, tumor invasion, and metastasis. It can also induce tumor cell apoptosis, regulate cell signal transduction, reverse drug resistance, and enhance sensitization to anti-tumor chemotherapeutic drugs (Ji et al., 2019). The anti-cancer effects of artesunate have been confirmed in liver cancer (Yao et al., 2020), ovarian cancer, and breast cancer cells (Zhang et al., 2018). Previous work in our laboratory confirmed that artesunate can regulate and inactivate MEK/ERK signaling, and inhibit the propagation of liver cancer cells. Because sorafenib does not directly act on the MEK/ERK signaling pathway, we speculate that artesunate may synergistically enhance the sensitivity of liver cancer cells to sorafenib.

Reticulons are a relatively newly characterized eukaryotic gene family, and they are widely distributed in plants, animals, and yeasts (Oertle et al., 2003). Neurite outgrowth inhibitor (Nogo) proteins, also known as reticulon-4, are myelin-associated glycoproteins. Three Nogo protein subtypes are generated via differences in the transcription and splicing process, Nogo-A, Nogo-B, and Nogo-C, which contain 1,192, 373, and 199 residues respectively. These three translational products differ but they are all distributed in the endoplasmic reticulum (Josephson et al., 2002). Nogo-B is a shorter subtype than Nogo-A, and is widely distributed in various tissues and organs of the human body, but not in the central nervous system. It is expressed in the liver, lung, kidney, blood vessels, pancreas, and inflammatory cells (Long et al., 2017). (Oertle and Schwab, 2003) first discovered the Nogo-B-specific receptor (NgBR). NgBR is widely distributed in various tissues and organs in the body (Duff et al., 2015), and has specific effects on nervous system regeneration, cell chemotaxis, apoptosis, and propagation. It also plays key roles in cell apoptosis, epithelial-mesenchymal transition, and the development of drug resistance in tumor cells. The Nogo-B gene, also known as ASY (Li et al., 2001), is a human apoptosis-inducing gene but it has no known specific apoptosis-related motif. One study has shown that Nogo-B overexpression promotes endoplasmic reticulum stress and intracellular calcium disorder, and ultimately induces apoptosis (Teng and Tang, 2013). Schweigreiter et al. (2007) have shown that Nogo-B can regulate cell apoptosis through the caspase-7 pathway. NgBR can reportedly also directly bind to isoamylated Ras, stabilizing it on the envelope and promoting its function, which in turn enhances epidermal growth factor signal pathway activity, promoting tumor formation (Zhao et al., 2017).

This study was designed to identify the relationship between neurite outgrowth inhibitor protein B receptor (NgBR) and sorafenib resistance. Our results show that artesunate can inhibit NgBR expression, and in combination sorafenib artesunate can overcome the resistance to sorafenib in hepatocellular carcinoma cells.

## Materials and Methods

### Cell Lines

The HepG2 human hepatoma cell line was purchased from the Shanghai Institute of Biochemistry and Cell Biology (Shanghai, China). All cell lines in the study were maintained in DMEM medium (Gibco, CA, United States) supplemented with 10% fetal bovine serum (Gibco, CA, United States), 50 μg/ml streptomycin, and 100 U/ml sodium penicillin at 37°C in an atmosphere containing 5% CO_2_. HepG2 sorafenib-resistant cells (HepG2-SR) were generated by selection starting with sorafenib (Selleckchem, United States) at 2 μm, increasing to 10 μm over the course of 3 months.

### Quantitative Real-Time PCR

TRIzol reagent was used to extract total cellular RNA in accordance with the manufacturer’s instructions (Thermo Fisher, United States). SYBR Green quantitative reverse transcription PCR (qRT-PCR) was used to evaluate NgBR mRNA expression levels. Primer NgBR F (5’-TGC​CAG​TTA​GTA​GCC​CAG​AAG​CAA-3’); NgBR R (5’-AAC​CTG​CCA​AGT​ATG​ATG​AC-3’)

### Cell Proliferation

The 3-(4,5-dimethylthiazol-2-yl)-2,5-diphenyltetrazolium bromide (MTT) assay and the 5-ethynyl-2’-deoxyuridine (EDU) immunofluorescence staining assay (Ribobio, Guangzhou, China) were used to test the propagation capacity of HCC cells. Sorafenib (99.15% purity) and artesunate (99.89% purity) were purchased from Selleck Chemical (United States). Briefly, for the MTT assay the cells were cultured at a density of 6,000 cells/well in 96-well plates in triplicate and treated with sorafenib and/or artesunate for 48 h. For the EDU immunofluorescence staining assay the cells were incubated with sorafenib for 24 h before permeabilization. EDU staining and fixation were performed in accordance with the manufacturer’s instructions. A concentration of 1 mg/ml of 4′,6-diamidino-2-phenylindole (DAPI; Beyotime, China) was used to stain the cell nuclei for 15 min. Cell numbers were calculated via confocal laser scanning microscopy (Olympus, FV10i, Japan).

### Colony Formation Assay

Cells were seeded into six-well plates (103 cells per well) and cultured in complete media for two weeks. After two weeks, cells were fixed with methanol (1%), stained with 1% crystal violet, and imaged using a digital camera (Olympus, Japan). Cell colonies (cell clone diameter ≥1 mm) were counted in this experiment.

### Fluorescein Isothiocyanate-Terminal Deoxynucleotidyl Transferase-Mediated dUTP Nick-End Labeling

Cells in logarithmic growth phase were seeded into culture flasks (1.5 × 10^5^ cells/ml) and treated with sorafenib and/or artesunate in complete DMEM for 48 h. Paraformaldehyde (4%) in phosphate-buffered saline was used to fix the cells for 30 min, followed by permeabilization in 0.3% Triton X-100 in phosphate-buffered saline for 5 min at room temperature. Cells were stained with terminal deoxynucleotidyl transferase-mediated dUTP nick-end labeling (TUNEL) reagent in the dark for 60 min, then the number of apoptotic cells was assessed via confocal laser scanning microscopy.

### Transfection

#### siRNA

Cells were plated in a 6-well culture plate for 24 h and transfection reagents prepared. The control group was treated with 2 ml transfection medium, 2 μl RNAiMAX, and 2 μl siNC. The siNgBR group was treated with 2 ml transfection medium, 2 μl RNAiMAX, and 2 μl siNgBR. The siNC and siNgBR group was transfected using transfection reagents in a 6-well culture plate. The siRNA sequences used were: siNgBR F (GGA​AAU​ACA​UAG​ACC​UAC​A), R (UGU​AGG​UCU​AUG​UAU​UUC​C); and siNC F (UUC​UCC​GAA​CGU​GUC​ACG​U), R (ACG​UGA​CAC​GUU​CGG​AGA​A) (QIAGEN).

#### Plasmids

NgBR overexpression plasmid transfection: cells were plated in 10-cm culture dishes for 24 h then 5 ml transfection medium, 20 μl Lipofectamine 2000 (Invitrogen), and 4 μg plasmid was prepared. Plasmid DNA and Lipofectamine 2000 were diluted in serum-free and antibiotic-free medium, respectively. Diluted DNA was added to diluted Lipofectamine 2000, mixed well, and incubated at room temperature for 20 min. NgBR overexpression group cells were transfected with the pIRES-NgBR-HA plasmid and control group cells were transfected with blank vector. The transfection efficiency was determined by western blot after 48 h incubation.

### Western Blotting

Radioimmunoprecipitation assay lysis buffer was used to prepare cell lysates for protein analysis. The BCA Assay Kit (Beyotime, China) was used to determine protein concentrations and achieve standardization among the samples. Aliquots of 40 μg of each lysate were prepared, run through SDS-PAGE, and transferred to a polyvinylidene difluoride membrane. The membrane was then blocked with 10% bovine serum albumin (Sigma, United States) and incubated at 4°C overnight with antibodies against the proteins of interest. The antibodies used were NgBR (ab168351, 1:1,000, Abcam, United Kingdom), Nogo-B (AF6034, 1:2,000, Imgenex, United States), phospho-Erk1/2 Thr202/Tyr204 (#4370, 1:1,000, Cell Signaling, United States), Erk1/2 p42/44 (#4696, 1:1,000, Cell Signaling), MEK1/2 (#8727, 1:1,000, Cell Signaling), phosphor-MEK1/2 Ser217/221 (#3958, 1:1,000, Cell Signaling), and β-actin (#3700, 1:1,000, Cell Signaling).

### Xenotransplantation Studies

All xenotransplantation experiments were approved by the Experimental Animal Ethics Committee of Jinzhou Medical University (protocol 20170503) and conformed to the Guide for the Care and Use of Laboratory Animals published by the United States National Institutes of Health (eighth Edition, 2011). Cells (1 × 10^7^) were resuspended in 100 μl DMEM and injected into the dorsal flanks of female 6-week-old nu/nu mice subcutaneously. Two weeks later, 20 mice with tumor volumes of approximately 100 mm^3^ were selected and dicided into four groups. The four group treatments were dimethylsulfoxide (DMSO) (Selleckchem, United States) with corn germ oil, artesunate (50 mg/kg) with corn germ oil, sorafenib (30 mg/kg) with corn germ oil, and artesunate/sorafenib (80 mg/kg/30 mg/kg) with corn germ oil. All treatments were administered once every 3 days for 21 days. The solvent used to dissolve the drugs was DMSO, with corn germ oil as a co-solvent.

### Data Analysis

All data were analyzed using GraphPad prism software 8.0. One-way analysis of variance and chi-square tests were used to compare single-factor groups. Inter-group analysis was performed using Student’s *t*-tests. *p* values <0.05 were considered statistically significant.

## Results

### Artesunate Inhibition of Hepatocarcinoma Parent Cell and Sorafenib Resistant Cell Proliferation

**FIGURE 1:**
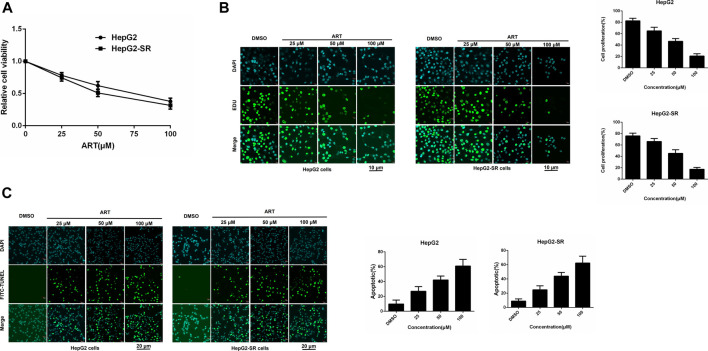
Artesunate (ART) can effectively inhibit the propagation of sorafenib-resistant cells. **(A)**. HepG2-SR cells were treated with 0, 25, 50, or 100 μm artesunate, and 48 h later MTT assays were performed to detect artesunate sensitivity in the cells. **(B)**. Cell propagation detected by EDU assays. Cells were stained with DAPI (blue) for visualization of nuclei, and EDU (green) signifies proliferative cells. EDU-positive cells per field were quantified in multiple fields, and cell proliferation was measured relative to total cells. Scale bars = 10 μm. **(C)**. Apoptosis detected via FITC-TUNEL staining assays. Blue (DAPI) represents nuclei, and green (FITC-TUNEL) represents apoptotic cells. FITC-TUNEL-positive cells per field were quantified in multiple fields and the ratio of apoptotic cells was measured relative to total cells. Scale bars = 20 μm. Experiments were repeated three times.

HepG2 cells and HepG2-SR cells were treated with 0, 25, 50, or 100 μm concentrations of artesunate for 48 h, and artesunate alone inhibited the propagation of HepG2 and HepG2-SR liver tumor cells in a dose-dependent manner (Figure 1A). Cell viability was investigated via EDU immunofluorescence staining assays after 24 h, and several concentrations of artesunate inhibited the growth of HepG2 and HepG2-SR cells (Figure 1B). FITC-TUNEL staining apoptosis assays also revealed dose-dependent artesunate-induced apoptosis of HepG2 and HepG2-SR cells (Figure 1C).

### Artesunate Inhibition of NgBR Expression in Sorafenib-Resistant HCC Cells

**FIGURE 2:**
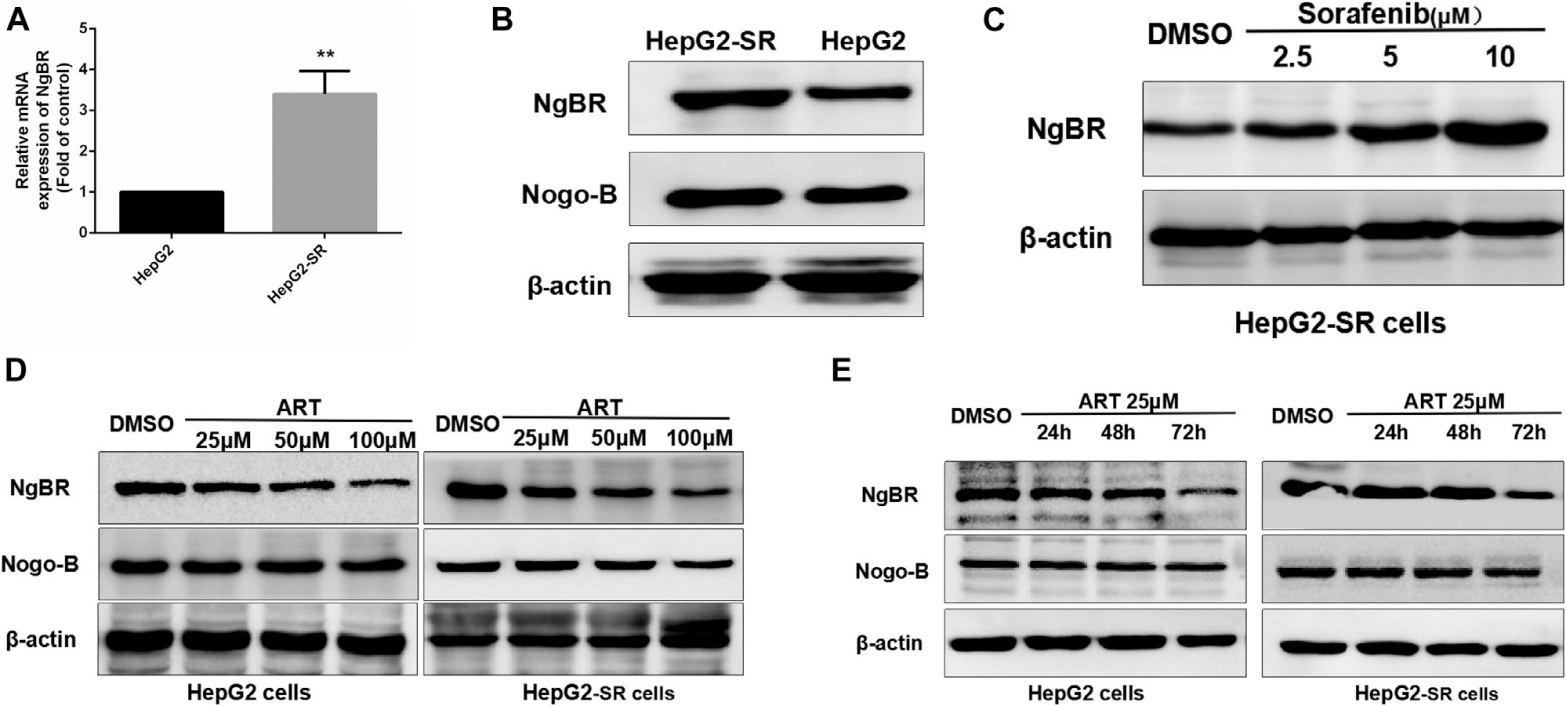
Artesunate (ART) can inhibit NgBR expression in sorafenib-resistant HCC cells. NgBR expression was higher in HepG2-SR cells as determined by **(A)** qRT-PCR and **(B)** western blotting. Western blotting **(C)** indicated that NgBR expression increased in a dose-dependent manner in the presence of sorafenib in HepG2-SR cells. The experiment was performed in triplicate. Western blotting indicated that artesunate can inhibit the expression of NgBR in both HepG2 cells and sorafenib-resistant HCC cells, in a dose-dependent manner **(D)** and in a time-dependent manner **(E)**. Experiments were repeated three times, and data are expressed as means ± the standard error of the mean. Student’s *t*-test was used to analyze the data. *p < 0.05

In qRT-PCR and western blotting analyses there were significant increases in NgBR expression in sorafenib-resistant cells compared to normal cells (Figurea 2A, B). Sorafenib affected NgBR expression in a dose-dependent manner in sorafenib-resistant cells (Figure 2C). In experiments in which sorafenib-resistant cells were treated with various concentrations of artesunate for 48 h or a uniform concentration for various times, artesunate reduced NgBR expression in a dose-dependent manner (Figure 2D) and a time-dependent manner (Figure 2E).

### NgBR Knockdown and the Restoration of Sorafenib Sensitivity in Sorafenib-Resistant Liver Cancer Cells

**FIGURE 3:**
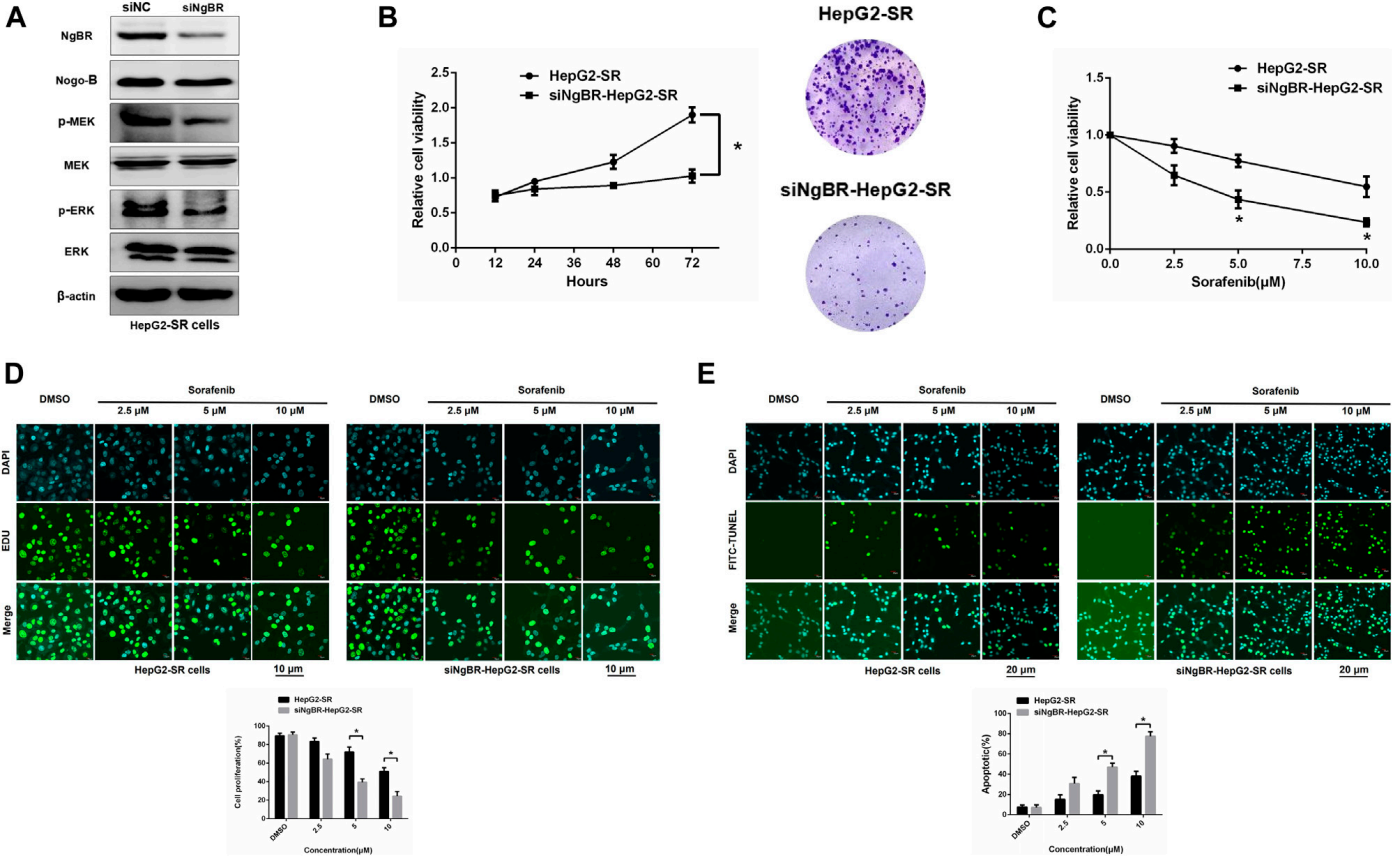
NgBR knockdown reduces sorafenib resistance. **(A)**. NgBR knockdown efficiency of was assessed via western blotting. **(B)**. Cell growth of NgBR knockdown and normal control HepG2-SR cells was measured by MTT (72 h) and colony formation (14 days) assays. Data are presented as means ± standard deviation (SD). **(C)**. HepG2-SR cells were treated with various doses (0, 2.5, 5, and 10 μm) of sorafenib. After 48 h, the MTT assay was used to detect sorafenib sensitivity. **(D)**. Cell propagation was detected by EDU assays. Scale bars, 10 μm. **(E)**. Apoptosis was detected by FITC-TUNEL staining. Scale bars, 20 μm. Experiments were repeated three times and data are presented as mean ± SD. Student’s *t*-test was used to analyze data. *p < 0.05

Western blotting was used to determine NgBR knockdown efficiency (Figure 3A). MTT (72 h) and colony formation (14 days) assays revealed that NgBR downregulation can reduce the viability of HepG2-SR cells (Figure 3B). Likewise, MTT (48 h) and EDU immunofluorescence staining (24 h) assays, after treatment with various sorafenib concentrations, revealed that NgBR knockdown significantly decreased cell proliferation in HepG2-SR cells. This effect was particularly evident with 5 and 10 μm sorafenib treatments (Figures 3C, D). FITC-TUNEL staining showed that sorafenib treatment at a lower concentrations (5 μm) caused significantly more apoptosis in NgBR-knockdown cells than in mock treated cells (Figure 3E).

### NgBR Overexpression in Hepatocarcinoma Parent Cells and the Promotion of Sorafenib Resistance in Liver Cancer Cells

**FIGURE 4:**
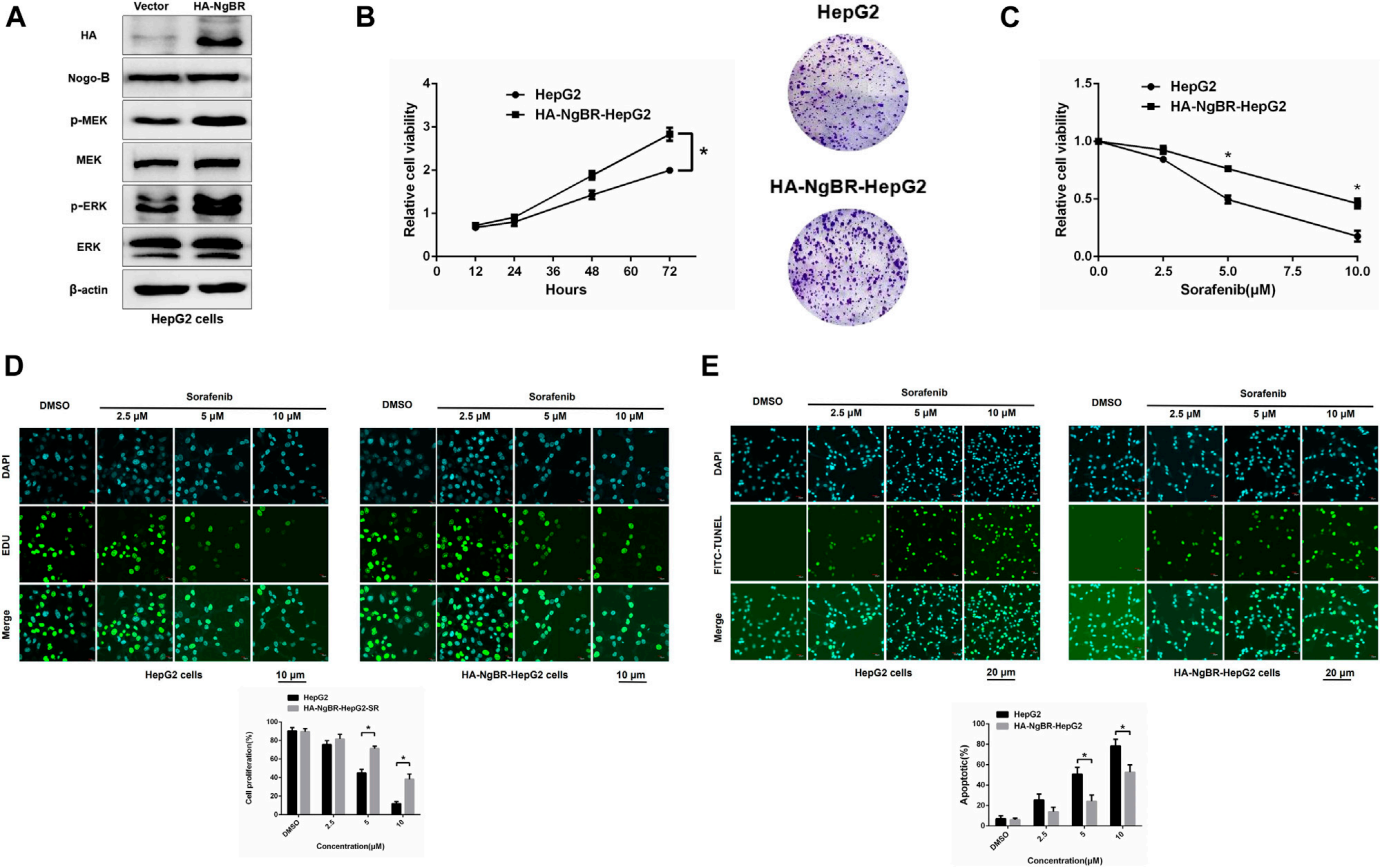
NgBR overexpression promotes sorafenib resistance in liver cancer cells. **(A)**. NgBR overexpression was assessed via western blotting. **(B)**. Cell growth in NgBR overexpression and normal control HepG2 cells was detected by MTT (72 h) and colony formation (14 days) assays. The data are presented as means ± standard deviation (SD). **(C)**. MTT assay was employed to evaluate cellular proliferation, as a measure of sorafenib sensitivity in the cells, after 48 h. **(D)**. Cell proliferation was detected by EDU assays. Scale bars, 10 μm. **(E)**. Apoptosis was detected by FITC-TUNEL staining assays. Scale bars, 20 μm. Experiments were repeated three times and data are presented as mean ± SD. Student’s *t*-test was used for data analysis. *p < 0.05

NgBR was overexpressed in parental HepG2 cells (Figure 4A). MTT (72 h) and colony formation (14 days) assays revealed that NgBR overexpression can increase HepG2 cell viability (Figure 4B). We also found that, in HepG2 cells, NgBR overexpression significantly decreases the sensitivity of liver cancer cells to sorafenib at doses of either 5 μm or 10 μm (Figures 4C, D), and decreases rates of apoptosis following sorafenib (5 μm or 10 μm) treatment (Figure 4E).

### Effects of Artesunate Combined With Sorafenib on Sorafenib Resistance *in vitro*

**FIGURE 5:**
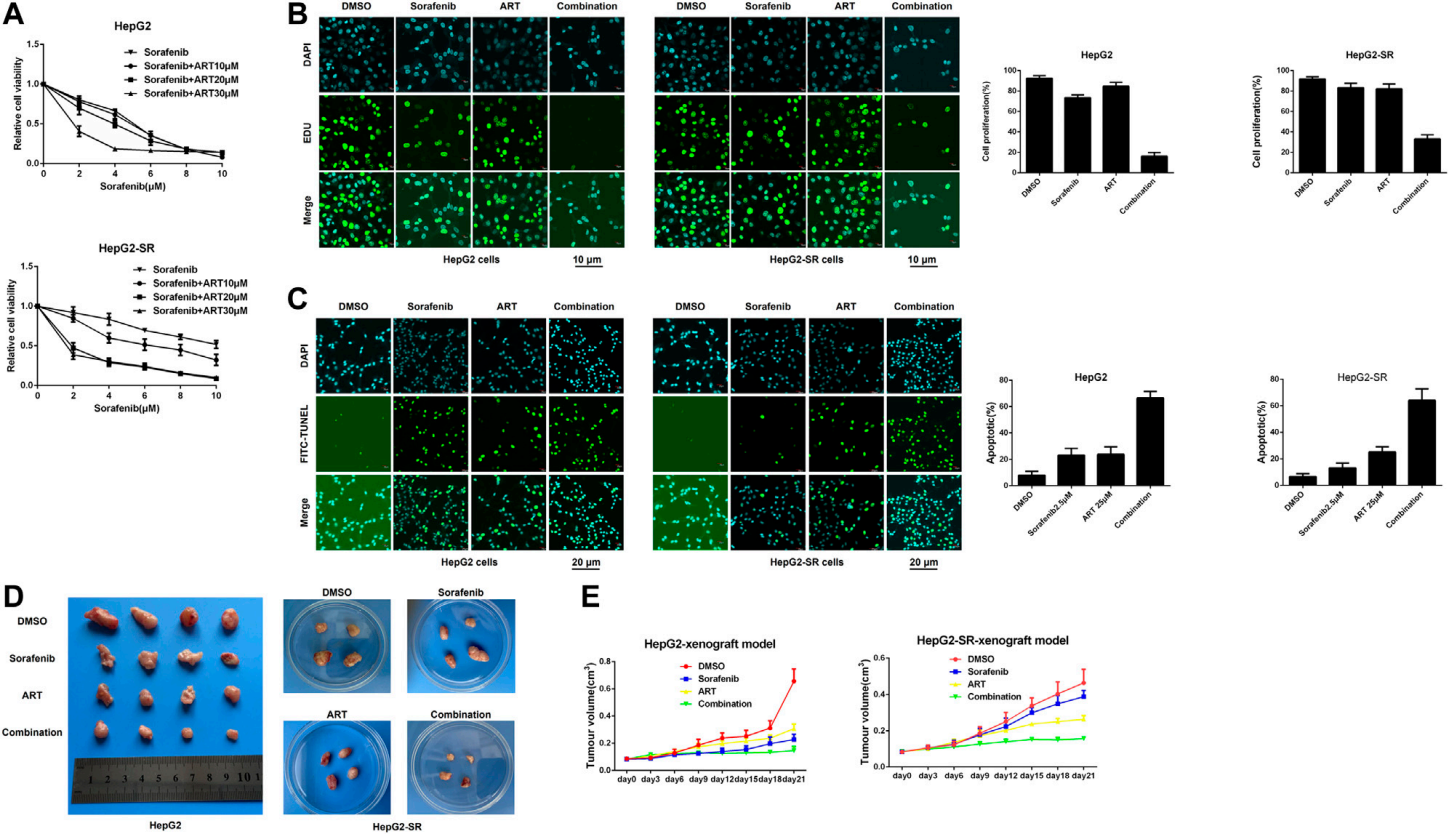
Artesunate (ART) combined with sorafenib overcomes adaptive resistance to sorafenib *in vitro* and *in vivo*. **(A)**. Cells were treated with sorafenib alone or in combination with a fixed dose of artesunate, then MTT assays were conducted after 48 h. **(B)**. Sorafenib and artesunate alone or in combination were used in HepG2 cells and HepG2-SR cells after 24 h, and cell proliferation was detected via EDU assays. Scale bars = 10 μm. **(C)**. HepG2-SR cells and HepG2 cells were treated with sorafenib and artesunate alone or in combination for 48 h, then apoptosis was detected via FITC-TUNEL staining assays. Scale bars = 20 μm. **(D)**. ∼1 × 107 HepG2 or HepG2-SR cells were injected into mouse flanks. When tumors reached ≥100 mm^3^ in size the mouse was administered (1) DMSO with corn germ oil, (2) sorafenib (30 mg/kg) with corn germ oil, (3) artesunate (50 mg/kg) with corn germ oil, or (4) sorafenib (30 mg/kg) and artesunate (50 mg/kg) with corn germ oil once every 3 days via oral cannular. Tumor sizes were measured periodically with calipers, and tumor volumes were calculated using the formula volume = (width^2^ × length)/2. **(E)**. After 21 days mice were killed by cervical dislocation, and the tumors were removed and weighed. Experiments were repeated three times.

The results of MTT assays conducted to determine appropriate concentrations of sorafenib alone or in combination with artesunate to achieve inhibitory effects on sorafenib-resistant liver cancer cells are shown in Figure 5A. In the combination study the sorafenib concentration was 2.5 μm and the artesunate concentration was 25 μm. HepG2-SR cells were treated with sorafenib (2.5 μm) and/or artesunate (25 μm) for 48 h, and artesunate rendered the cells significantly more sensitive to sorafenib. Inhibited proliferation and increased apoptosis were associated with sorafenib sensitivity (Figures 5B, C).

### Effects of Artesunate Combined With Sorafenib on Sorafenib Resistance *in vivo*

In experiments using xenografts derived from HepG2 and HepG2-SR cells to examine efficacy *in vivo*, after 3 weeks sorafenib alone and artesunate alone inhibited cancer growth as measured by tumor weight. The combination of artesunate and sorafenib resulted in greater growth inhibition than artesunate or sorafenib alone in both HepG2 and HepG2-SR xenograft models (Figures 5D, E). Thus, artesunate combined with sorafenib overcame NgBR-dependent sorafenib resistance in liver tumors *in vivo*.

### Artesunate can Block NgBR-Induced Sorafenib Resistance by Suppressing the MEK/ERK Pathway

**FIGURE 6:**
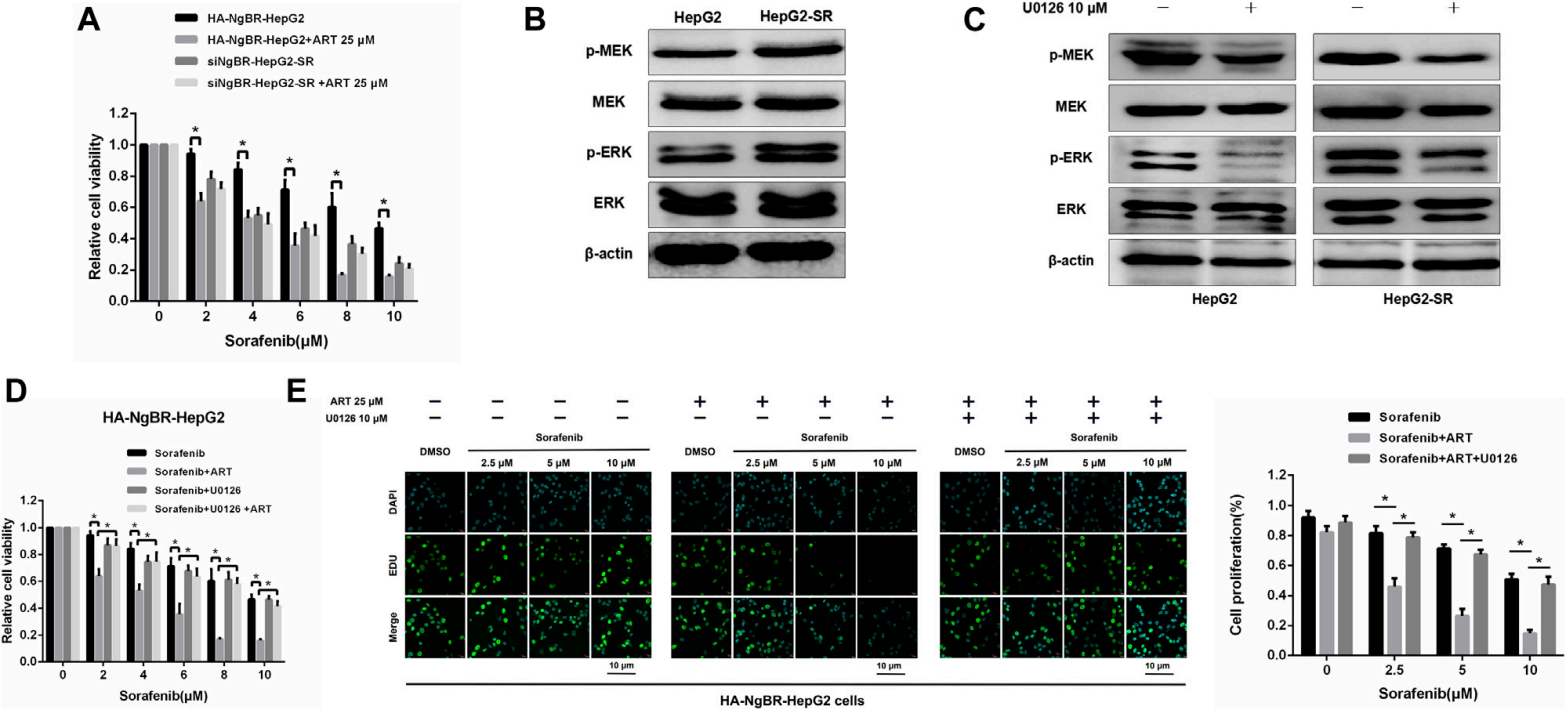
Artesunate (ART) overcomes NgBR-induced sorafenib resistance by suppressing the MEK/ERK pathway. **(A)**. MTT assay detected proliferation in HepG2-SR cells with NgBR downregulation and HepG2 cells with NgBR overexpression after treatment with sorafenib alone or in combination with artesunate. **(B)**. Phosphorylation levels of MEK (p-MEK) and ERK (p-ERK) in HepG2 and HepG2-SR cells were detected by western blot. **(C)**. HepG2 and HepG2-SR cells were treated with 10 μm U0126 for 7 days to inhibit p-MEK and p-ERK. Western blot was used to determine inhibition efficiency. **(D)**. HepG2 cells with NgBR overexpression were treated with 10 μm U0126 for 7 days, and MTT assays were used to detect the artesunate-induced sorafenib sensitization. **(E)**. EDU assays were used to detect the proliferation in HepG2 cells overexpressing NgBR and treated with 10 μm U0126 for 7 days. Scale bars, 10 μm. Experiments were repeated three times and data are presented as means ± standard deviation (SD). Student’s *t*-test was used for data analysis. *p < 0.05

The role of NgBR downregulation and overexpression in artesunate-induced sorafenib sensitization was examined. Firstly, The MTT assay was used to determine the appropriate concentrations of sorafenib alone, or in combination with artesunate, to use in HepG2-SR cells with downregulated NgBR and HepG2 cells with NgBR overexpression (Figure 6A). We found that artesunate can effectively inhibit cell proliferation in NgBR overexpressing cells only. To confirm that NgBR promotes HepG2 cell proliferation and survival through the activation of MEK/ERK signaling we examined the expression of relevant proteins. Activation of MEK/ERK signaling in HepG2 and HepG2-SR cells was examined by western blot. We found that, compared to HepG2 cells, the phosphorylation levels of MEK (p-MEK) and ERK (p-ERK) were significantly upregulated in HepG2-SR cells (Figure 6B). We then treated HepG2 and HepG2-SR cells with 10 μm U0126, a MEK 1/inhibitor, for 7 days. Western blotting performed after U0126 treatment indicated that U0126 can inhibit p-MEK and p-ERK levels in both HepG2 and HepG2-SR cells (Figure 6C). MTT and EDU immunofluorescence staining (24 h) assays showed that in NgBR overexpressing HepG2 cells, artesunate-induced sorafenib sensitization was significantly decreased in U0126-treated cellscompared to U0126-untreated cells (Figures 6D, E).

## Discussion

Sorafenib is a molecularly targeted drug available for the clinical management of liver cancer, but the development of resistance to sorafenib in liver cancer cells has reduced its therapeutic efficacy. Therefore, the current study investigated the mechanism of resistance of liver cancer to sorafenib. Increasing the cancer cells’ sensitivity to therapeutic molecules is one of the main strategies for effective liver cancer treatment. Artesunate is a widely used effective anti-malarial agent, but studies have also confirmed that it has anti-tumor effects (Ye et al., 2020). Moreover, its cost-effectiveness and comparatively fewer adverse effects make it an ideal anti-tumor drug. The *in vitro* experimental results of the present study indicate that artesunate can prevent the propagation of hepatocarcinoma parent cells as well as cells resistant to sorafenib in a dose-dependent manner, and promote their apoptosis.

Previous studies suggest that NgBR upregulation may affect the occurrence and development of hormone receptor-positive breast cancer (Wang et al., 2013). Studies investigating tamoxifen resistance also indicate that epithelial-mesenchymal transition can play a key role (Liang et al., 2017). NgBR promotes epithelial-mesenchymal transition in breast and lung cancer (Zhao et al., 2015; Wu et al., 2018). The role of NgBR in the development of resistance to chemotherapy in HCC and breast cancer is clear, but whether NgBR can affect the development of sorafenib resistance has not been reported.

In this study, we investigated the correlation between NgBR and sorafenib resistance in HCC. To examine the correlation between sorafenib resistance and NgBR, we developed a sorafenib-resistant HCC cell line for *in vitro* studies. All sorafenib-resistant liver cancer cells showed strong propagation and reduced apoptosis in the presence of sorafenib. Additionally, NgBR was overexpressed in sorafenib-resistant cells, and sorafenib promoted NgBR expression in sorafenib-resistant liver cancer cells in a dose-dependent fashion. To study the role of NgBR in developing resistance to sorafenib, we knocked down NgBR in sorafenib-resistant cells. Sorafenib sensitivity increased in HepG2-SR knockdown cells, but not in mock-treated HepG2-SR cells. Then, we overexpressed NgBR in HCC parent cells and found that NgBR overexpression accounted for the observed sorafenib resistance in HCC. Therefore, we concluded that NgBR expression in sorafenib-resistant cells was correlated with resistance to sorafenib.

According to previous studies, artesunate can promote apoptosis and inhibit the propagation of sorafenib-resistant liver cancer cells. We speculated that artesunate functioned through inhibiting NgBR. Time and concentration-dependent studies confirmed that artesunate can inhibit the NgBR expression in sorafenib-resistant liver cancer cells.

Artesunate prevented the progression of sorafenib resistance in liver cancer cells by inhibiting NgBR expression, suggesting that artesunate, in combination with sorafenib, can increase the sensitivity of liver tumor cells to sorafenib. This study confirmed that artesunate can enhance the sensitivity of liver cancer cells to sorafenib both *in vivo* and *in vitro*. HepG2 cells overexpressing NgBR and HepG2-SR cells with downregulated NgBR were treated with different concentrations of sorafenib alone or in combination with artesunate. We found that artesunate can only effectively inhibit cell proliferation in NgBR overexpressing cells. This indicates that artesunate enhances the sensitivity of liver cancer cells to sorafenib by inhibiting the NgBR expression.

In a previous study, Gao et al. discovered that increased NgBR can enhance EGF-stimulated Ras activation and phosphorylation of AKT and ERK in tamoxifen-resistant breast cancer cells (Gao et al., 2018). Moreover, NgBR expression is associated with non-small cell lung cancer (NSCLC) development and increases Snail 1 expression in NSCLC cells by MEK/ERK pathway activation (Wu.et al., 2018). We examined the MRK/ERK pathway using western blot analysis. NgBR expression was positively correlated with the activation of phosphorylated MEK, confirming that the MEK/ERK pathway was significantly upstream in HepG2-SR cells. U0126-EtOH, a highly selective MEK1/2 inhibitor, was used to treat NgBR overexpressing HepG2 cells for 7 days. Compared to the non-treated U0126-EtOH group, the effects of artesunate-induced sorafenib sensitivity were inhibited by the MEK1/2 inhibitor. This result indicates that NgBR promotes HepG2 cell proliferation and survival through MEK/ERK pathway activation, and that artesunate can overcome NgBR-induced sorafenib resistance by suppressing the MEK/ERK pathway. The main limitation of this study is that it does not investigate the specific mechanism through which artesunate inhibits NgBR to increase the sensitivity of liver cancer cells to sorafenib. Thus, further studies focusing on the specific mechanism should be performed.

Taken together, the results of the current study demonstrate that NgBR is highly expressed in sorafenib-resistant HCC cells. NgBR is a functional marker that is essential for sorafenib resistance via activation of the MEK/ERK pathway. Artesunate can increase liver cancer cell sensitivity to sorafenib via inhibition of NgBR. Thus, targeting NgBR with a combination of artesunate and sorafenib can effectively overcome sorafenib resistance in HCC cells.

## Data Availability

The original contributions presented in the study are included in the article/Supplementary Material, further inquiries can be directed to the corresponding author.

## References

[B1] Al-Salama Z. T. Syed Y. Y. Scott L. J. 2019). Lenvatinib: a review in hepatocellular carcinoma. Drugs 79 (6), 665–674. 10.1007/s40265-019-01116-x 30993651

[B2] Díaz-González Á. Sanduzzi-Zamparelli M. da Fonseca L. G. Di Costanzo G. G. Alves R. Iavarone M. (2020). International and multicenter real-world study of sorafenib-treated patients with hepatocellular carcinoma under dialysis. Liver Int. 40 (6), 1467–1476. 10.1111/liv.14436 32170821

[B3] Duff M. O. Olson S. Wei X. Garrett S. C. Osman A. Bolisetty M. (2015). Genome-wide identification of zero nucleotide recursive splicing in *Drosophila* . Nature 521 (7552), 376–379. 10.1038/nature14475 25970244 PMC4529404

[B4] Fukui N. Golabi P. Otgonsuren M. de Avila L. Bush H. Younossi Z. M. (2018). Hospice care in Medicare patients with primary liver cancer: the impact on resource utilisation and mortality. Aliment. Pharmacol. Ther. 47 (5), 680–688. 10.1111/apt.14484 29314093

[B5] Gao P. Wang X. Jin Y. Hu W. Duan Y. Shi A. (2018). Nogo-B receptor increases the resistance to tamoxifen in estrogen receptor-positive breast cancer cells. Breast Cancer Res. 20 (1), 112. 10.1186/s13058-018-1028-5 30208932 PMC6134690

[B6] Ghousein A. Mosca N. Cartier F. Charpentier J. Dupuy J. W. Raymond A. A. (2020). miR-4510 blocks hepatocellular carcinoma development through RAF1 targeting and RAS/RAF/MEK/ERK signalling inactivation. Liver Int. 40 (1), 240–251. 10.1111/liv.14276 31612616

[B7] Gramantieri L. Pollutri D. Gagliardi M. Giovannini C. Quarta S. Ferracin M. (2020). MiR-30e-3p influences tumor phenotype through MDM2/TP53 *Axis* and predicts sorafenib resistance in hepatocellular carcinoma. Cancer Res. 80 (8), 1720–1734. 10.1158/0008-5472.CAN-19-0472 32015093

[B8] Hashiba T. Yamashita T. Okada H. Nio K. Hayashi T. Asahina Y. (2020). Inactivation of transcriptional repressor capicua confers sorafenib resistance in human hepatocellular carcinoma. Cell Mol. Gastroenterol. Hepatol. 10 (2), 269–285. 10.1016/j.jcmgh.2020.02.009 32169577 PMC7305345

[B9] Ji P. Huang H. Yuan S. Wang L. Wang S. Chen Y. (2019). ROS-mediated apoptosis and anticancer effect achieved by artesunate and auxiliary Fe(II) released from ferriferous oxide-containing recombinant apoferritin. Adv. Healthc. Mater. 8 (23), e1900911. 10.1002/adhm.201900911 31701665

[B10] Josephson A. Trifunovski A. Widmer H. R. Widenfalk J. Olson L. Spenger C. (2002). Nogo-receptor gene activity: cellular localization and developmental regulation of mRNA in mice and humans. J. Comp. Neurol. 453 (3), 292–304. 10.1002/cne.10408 12378589

[B11] Li Q. Qi B. Oka K. Shimakage M. Yoshioka N. Inoue H. (2001). Link of a new type of apoptosis-inducing gene ASY/Nogo-B to human cancer. Oncogene 20 (30), 3929–3936. 10.1038/sj.onc.1204536 11494121

[B12] Liang Y. K. Zeng D. Xiao Y. S. Wu Y. Ouyang Y. X. Chen M. (2017). MCAM/CD146 promotes tamoxifen resistance in breast cancer cells through induction of epithelial-mesenchymal transition, decreased ERα expression and AKT activation. Cancer Lett. 386, 65–76. 10.1016/j.canlet.2016.11.004 27838413

[B13] Long S. L. Li Y. K. Xie Y. J. Long Z. F. Shi J. F. Mo Z. C. (2017). Neurite outgrowth inhibitor B receptor: a versatile receptor with multiple functions and actions. DNA Cell Biol. 36 (12), 1142–1150. 10.1089/dna.2017.3813 29058484

[B14] Oertle T. Huber C. van der Putten H. Schwab M. E. (2003). Genomic structure and functional characterisation of the promoters of human and mouse nogo/rtn4. J. Mol. Biol. 325 (2), 299–323. 10.1016/s0022-2836(02)01179-8 12488097

[B15] Oertle T. Schwab M. E. (2003). Nogo and its paRTNers. Trends Cell Biol. 13 (4), 187–194. 10.1016/s0962-8924(03)00035-7 12667756

[B16] Rudalska R. Dauch D. Longerich T. McJunkin K. Wuestefeld T. Kang T. W. (2014). *In vivo* RNAi screening identifies a mechanism of sorafenib resistance in liver cancer. Nat. Med. 20 (10), 1138–1146. 10.1038/nm.3679 25216638 PMC4587571

[B17] Schweigreiter R. Stasyk T. Contarini I. Frauscher S. Oertle T. Klimaschewski L. (2007). Phosphorylation-regulated cleavage of the reticulon protein Nogo-B by caspase-7 at a noncanonical recognition site. Proteomics 7 (24), 4457–4467. 10.1002/pmic.200700499 18072206

[B18] Sung Y. C. Liu Y. C. Chao P. H. Chang C. C. Jin P. R. Lin T. T. (2018). Combined delivery of sorafenib and a MEK inhibitor using CXCR4-targeted nanoparticles reduces hepatic fibrosis and prevents tumor development. Theranostics 8 (4), 894–905. 10.7150/thno.21168 29463989 PMC5817100

[B19] Teng F. Y. Tang B. L. (2013). Nogo/RTN4 isoforms and RTN3 expression protect SH-SY5Y cells against multiple death insults. Mol. Cell. Biochem. 384 (1-2), 7–19. 10.1007/s11010-013-1776-6 23955438

[B20] Wang B. Zhao B. North P. Kong A. Huang J. Miao Q. R. (2013). Expression of NgBR is highly associated with estrogen receptor alpha and surviving in breast cancer. PloS One 8 (11), e78083. 10.1371/journal.pone.0078083.e 24223763 PMC3817177

[B21] Wang H. Xu H. Ma F. Zhan M. Yang X. Hua S. (2020). Zinc finger protein 703 induces EMT and sorafenib resistance in hepatocellular carcinoma by transactivating CLDN4 expression. Cell Death Dis. 11 (4), 225. 10.1038/s41419-020-2422-3 32269215 PMC7142083

[B22] Wu D. Zhao B. Qi X. Peng F. Fu H. Chi X. (2018). Nogo-B receptor promotes epithelial-mesenchymal transition in non-small cell lung cancer cells through the Ras/ERK/Snail1 pathway. Cancer Lett. 418, 135–146. 10.1016/j.canlet.2018.01.030 29331415 PMC7385903

[B23] Yao X. Zhao C.-r. Yin H. Wang K. Gao J.-j. (2020). Synergistic antitumor activity of sorafenib and artesunate in hepatocellular carcinoma cells. Acta Pharmacol. Sin. 41, 1609–1620. 10.1038/s41401-020-0395-5 32300243 PMC7921114

[B24] Ye R. R. Peng W. Chen B. C. Jiang N. Chen X. Q. Mao Z. W. (2020). Mitochondria-targeted artesunate conjugated cyclometalated iridium(iii) complexes as potent anti-HepG2 hepatocellular carcinoma agents. Metallomics 12 (7), 1131–1141. 10.1039/d0mt00060d 32453319

[B25] Zhang S. Gao W. Tang J. Zhang H. Zhou Y. Liu J. (2020). The roles of GSK-3β in regulation of retinoid signaling and sorafenib treatment response in hepatocellular carcinoma. Theranostics 10 (3), 1230–1244. 10.7150/thno.38711 31938062 PMC6956800

[B26] Zhang S. Yuan H. Guo Y. Wang K. Wang X. Guo Z. (2018). Towards rational design of RAD51-targeting prodrugs: platinum IV-artesunate conjugates with enhanced cytotoxicity against BRCA-proficient ovarian and breast cancer cells. Chem. Commun. 54 (83), 11717–11720. 10.1039/c8cc06576d 30229259

[B27] Zhao B. Hu W. Kumar S. Gonyo P. Rana U. Liu Z. (2017). The Nogo-B receptor promotes Ras plasma membrane localization and activation. Oncogene 36 (24), 3406–3416. 10.1038/onc.2016.484 28068323 PMC5472485

[B28] Zhao B. Xu B. Hu W. Song C. Wang F. Liu Z. (2015). Comprehensive proteome quantification reveals NgBR as a new regulator for epithelial-mesenchymal transition of breast tumor cells. J. Proteomics. 112, 38–52. 10.1016/j.jprot.2014.08.007 25173099 PMC4312238

